# Supplementary material for: SARS-CoV-2 infection induces inflammatory bone loss in golden Syrian hamsters
Source: Nat Commun. 2022 May 9;13:2539. doi: 10.1038/s41467-022-30195-w (PMC9085785; doi:10.1038/s41467-022-30195-w)
Supplement: Supplementary file 1 — Supplementary information [file 41467_2022_30195_MOESM1_ESM.pdf]

Supplementary Information

SARS-CoV-2 infection induces inflammatory bone loss in golden Syrian hamsters

Wei Qiao [1,2,3], Hui En Lau [1], Huizhi Xie [1,2], Vincent Kwok-Man Poon [4,5], Chris Chung-Sing Chan [4,5], Hin Chu [4,5,6], Shuofeng Yuan [4,5,6], Terrence Tsz-Tai Yuen [4,5], Kenn Ka-Heng Chik [4,5], Jessica Oi-Ling Tsang [4,5], Chris Chun-Yiu Chan [4,5], Jian-Piao Cai [4,5], Cuiting Luo [4,5], Kwok-Yong Yuen [4,5,6,7], Kenneth Man-Chee Cheung [1], Jasper Fuk-Woo Chan [4,5,6,7,8,✉], Kelvin Wai-Kwok Yeung [1,2,✉]

[1]Department of Orthopaedics and Traumatology, School of Clinical Medicine, Li Ka Shing Faculty of Medicine, the University of Hong Kong, Hong Kong S.A.R., China

[2]Shenzhen Key Laboratory for Innovative Technology in Orthopaedic Trauma, the University of Hong Kong-Shenzhen Hospital, Shenzhen 518053, China

[3]Applied Oral Sciences & Community Dental Care, Faculty of Dentistry, the University of Hong Kong, Hong Kong S.A.R., China

[4]State Key Laboratory of Emerging Infectious Diseases, School of Clinical Medicine, Li Ka Shing Faculty of Medicine, The University of Hong Kong, Pokfulam, Hong Kong Special Administrative Region, China.

[5]Department of Microbiology and Carol Yu Centre for Infection, School of Clinical Medicine, Li Ka Shing Faculty of Medicine, The University of Hong Kong, Pokfulam, Hong Kong Special Administrative Region, China.

[6]Department of Clinical Microbiology and Infection Control, The University of Hong Kong-Shenzhen Hospital, Shenzhen, Guangdong Province, China.

[7]Academician Workstation of Hainan Province of Hainan Medical University, and Hainan Medical University-The University of Hong Kong Joint Laboratory of Tropical Infectious Diseases, China.

[8]Guangzhou Laboratory, Guangdong Province, China.

✉ **Corresponding authors:** jfwchan@hku.hk (J.F.-W.C.) and wkkyeung@hku.hk (K.W.-K.Y.)

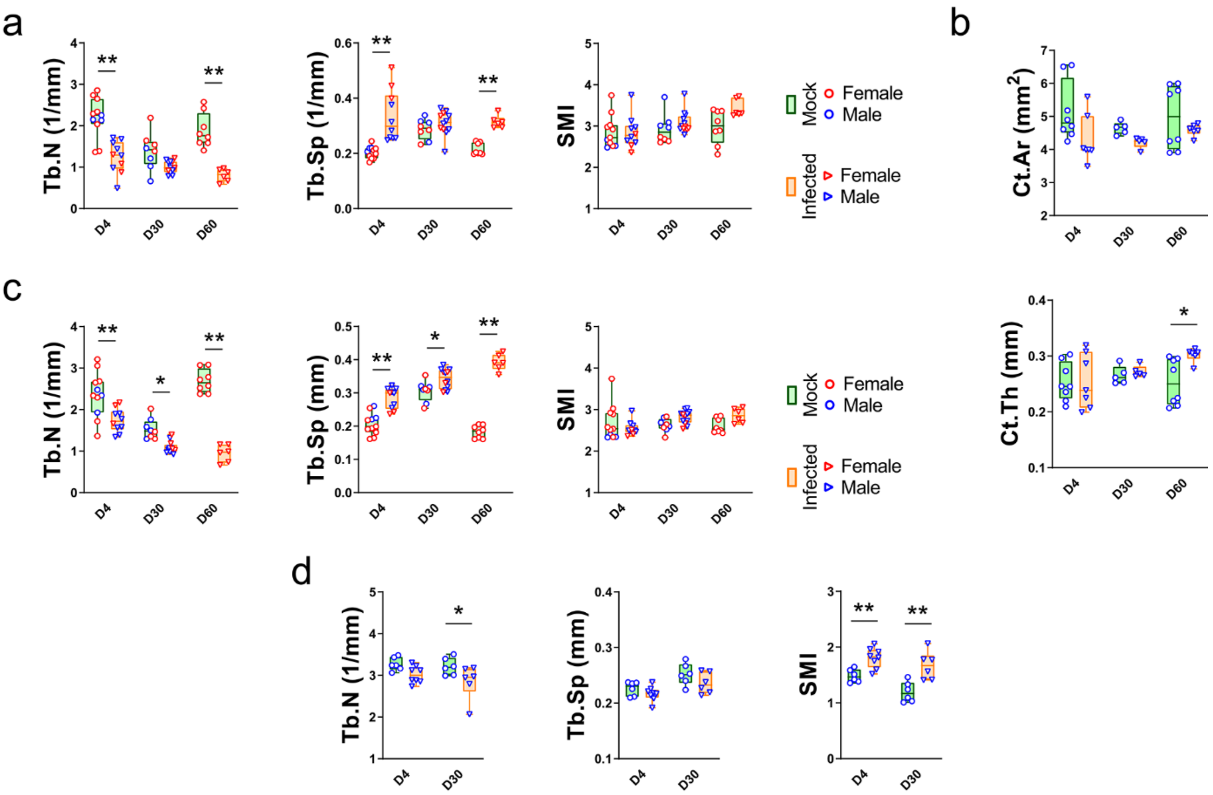

Supplementary Fig. 1: (a) µCT measurements of trabecular thickness (Tb.N), trabecular separation (Tb.Sp), and structure model index (SMI) of femurs. Mock: n =11 (D4), 8 (D30), 8 (D60); Infected: n =10 (D4), 12 (D30), 6 (D60). **(b)** µCT measurements of cortical bone area (Ct.Ar) and cortical bone thickness (Ct.Th) of femurs. Mock: n =8 (D4), 5 (D30), 8 (D60); Infected: n =7 (D4), 5 (D30), 6 (D60). **(c)** µCT measurements of trabecular thickness (Tb.N), trabecular separation (Tb.Sp), and structure model index (SMI) of tibias. Mock: n =11 (D4), 8 (D30), 8 (D60); Infected: n =10 (D4), 12 (D30), 6 (D60). **(d)** µCT measurements of trabecular thickness (Tb.N), trabecular separation (Tb.Sp), and structure model index (SMI) of lumbar vertebrae. Mock: n =6 (D4), 6 (D30); Infected: n =8 (D4), 6 (D30). Data are presented as box plots with whiskers from minima to maxima, the central line at the 50th percentile, and the ends of the box at the 25th and 75th percentiles. *P<0.05 and **P<0.01 by 2-sided Student's T-test **(c)**.

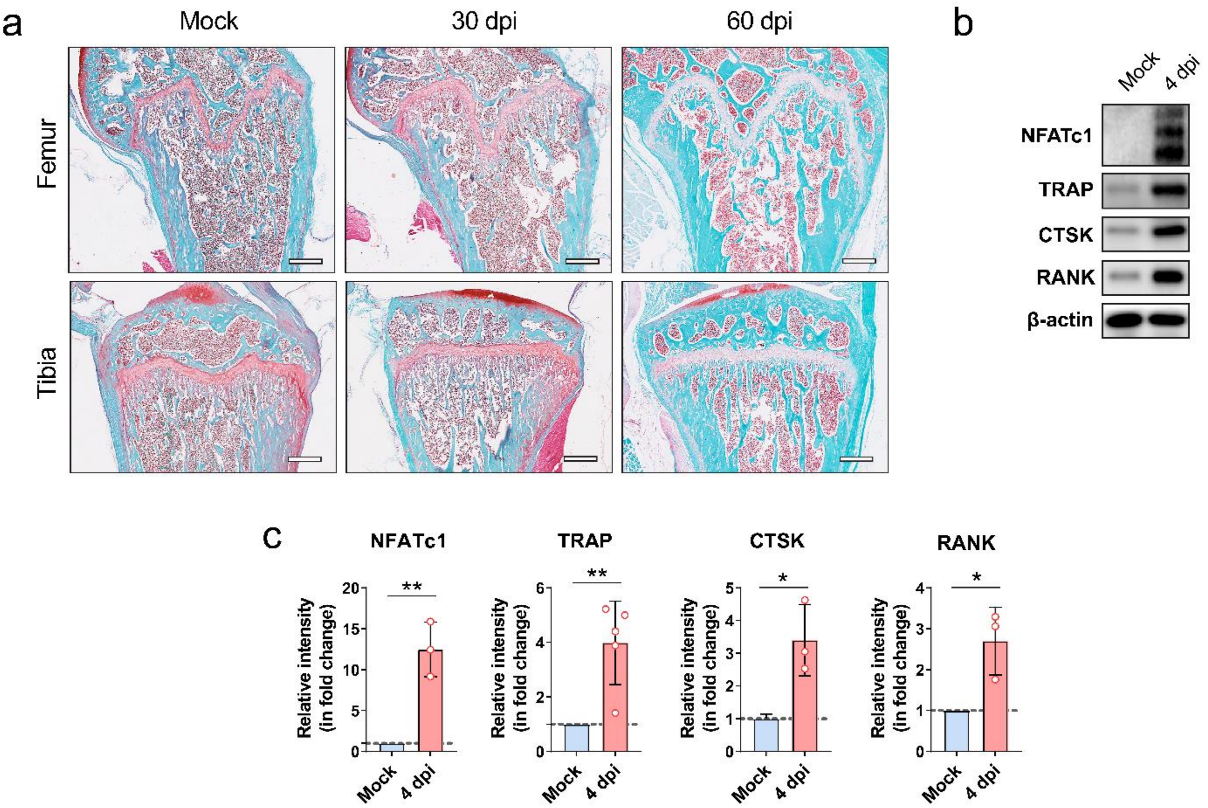

Supplementary Fig. 2: (a) Representative safranin O staining images showing the femurs and tibias of golden Syrian hamster after the infection of SARS-CoV-2 (n=4, scale bars = 500 μm). **(b, c)** Representative Western blots **(b)** and corresponding quantification **(c)** showing the increase in osteoclastic markers, including NFATc1 (n=3), TRAP (n=5), CTSK (n=3), and RANK (n=3) after the infection of SARS-CoV-2. Data are mean ± SD. **P*<0.05 and ***P*<0.01 by 2-sided Student's T-test **(c)**.

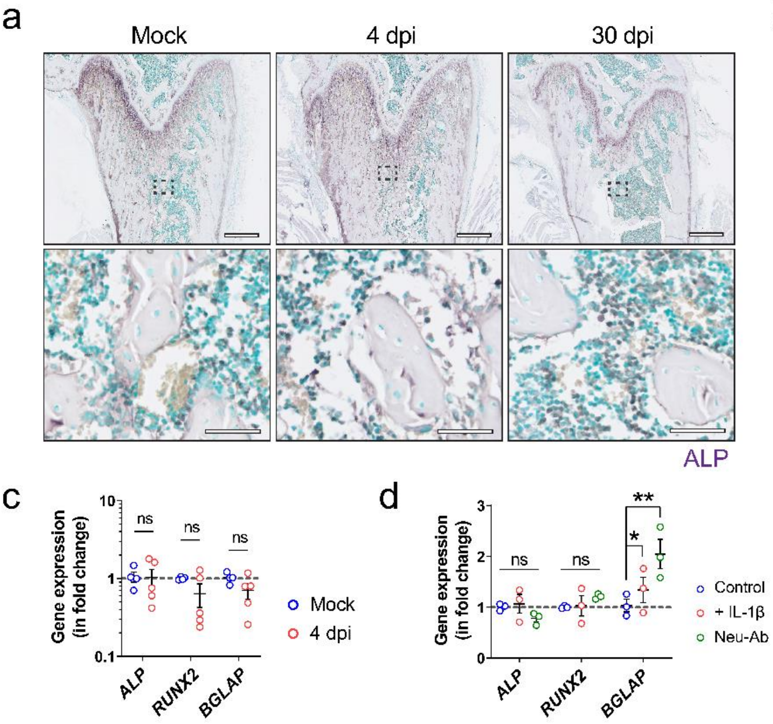

Supplementary Fig. 3: (a) Representative ALP staining images showing the osteoblastic activity in femurs of golden Syrian hamster after the infection of SARS-CoV-2. Lower images (scale bars = 100 µm) are high-resolution versions of the boxed regions in the upper images (scale bars = 500 µm). **(b)** Representative immunofluorescent images showing the OCN^{+} osteoblast at the distal metaphysis of femur after the infection of SARS-CoV-2. Tile scans (scale bars = 200 µm) and high-magnification of the boxed regions (scale bars = 50 µm) are shown. **(c)** The expression of osteogenesis-related genes in bone tissue at day 4 after being challenged with SARS-CoV-2 (n=5) or PBS (n=4). **(d)** The expression of osteogenesis-related genes in MSC with or without the presence of murine recombinant IL-1β and its neutralizing antibody (n=3). Data are mean ± SEM. ns: P>0.05, *P<0.05, **P<0.01 by 2-sided Student's T-test **(c)** or one-way ANOVA with Tukey's *post hoc* test **(d)**.

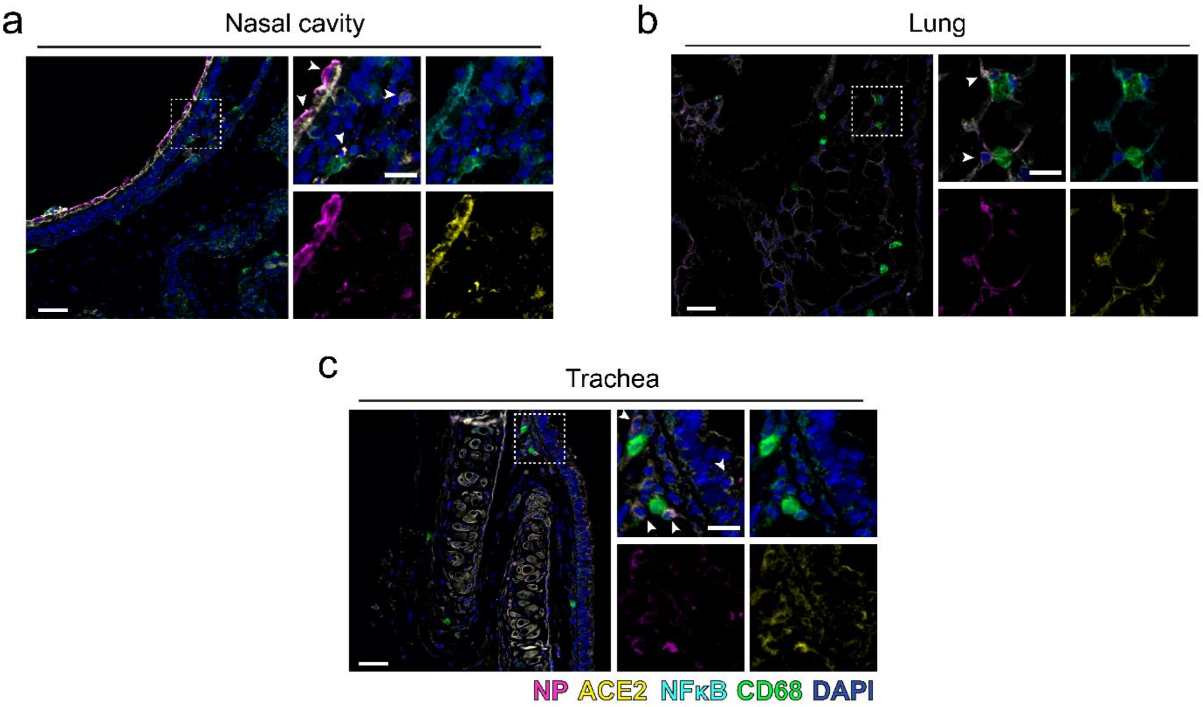

Supplementary Fig. 4: (a-c) Representative multi-color IHC (n=3) for Viral N protein (NP), Angiotensin-converting enzyme 2 (ACE2), NF-κB p65, and CD68 was performed at the epithelium of nasal cavity **(a)**, lung **(b)**, and trachea **(c)** on day four after the golden Syrian hamster was challenged with PBS (Mock) or SARS-CoV-2 (4 dpi). DAPI was used for nuclear counterstaining. Tile scans (scale bars = 200 μm) and high-magnification of the boxed regions (scale bars = 50 μm) are shown. White arrowhead indicated the virus infected cells expressing ACE2.

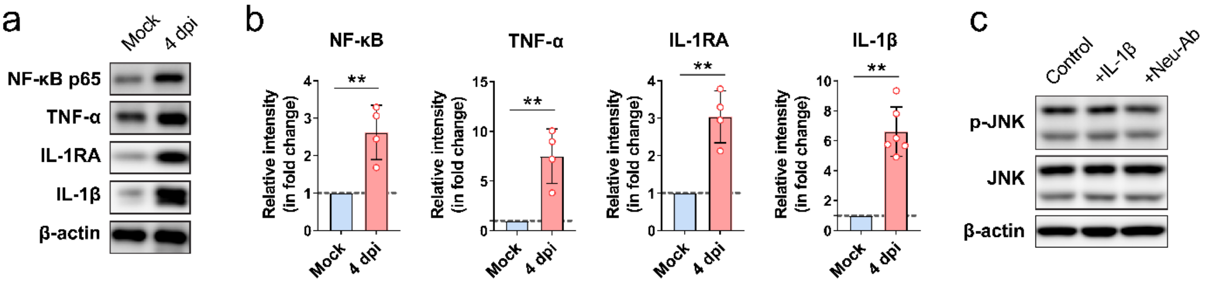

Supplementary Fig. 5: (a,b) Representative Western blots **(a)** and corresponding quantification **(b)** showing the expression of NF-κB (n=4), TNF-α (n=4), IL-1RA (n=4), and IL-1β (n=6), in the bone tissue of hamsters after the infection of SARS-CoV-2. **(c)** Representative Western blots showing the phosphorylated JNK after the addition of murine recombinant IL-1β or its neutralizing antibody (Neu-Abs). Data are mean ± SEM. *P<0.05 and **P<0.01 by 2-sided Student's T-test **(b)**.

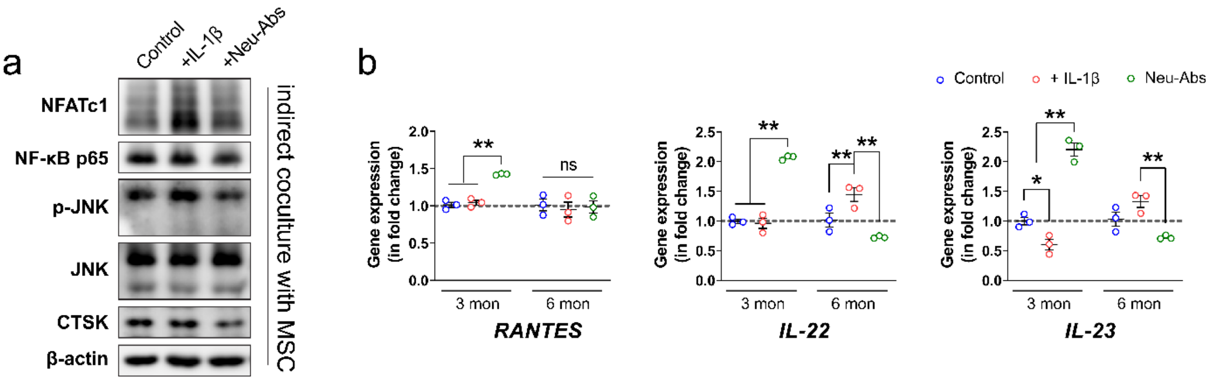

Supplementary Fig. 6: (a) Representative western blots showing the expression of NFATc1, NF-κB p65, and CTSK, as well as the phosphorylation of JNK after co-cultured with MSC treated with murine recombinant IL-1β or its neutralizing antibody. **(b)** The expression of inflammation-related genes in BMMs from young or adult mice with or without the addition of recombinant murine IL-1β or its neutralizing antibody (n=3). Data are mean ± SEM. ns: $P>0.05$, *$P<0.05$, **$P<0.01$ by one-way ANOVA with Tukey's *post hoc* test **(b)**.

Supplementary table 1. Primers used for RT-qPCR analysis of mRNA expression in hamster specimens

Genes	Primer sequences
IL-21	Forward: 5'- GGA CAG TGG CCC ATA AAA CAA G -3'
	Reverse: 5'- TTC AAC ACT GTC TAT AAG ATG ACG AAG TC -3'
IFN-γ	Forward: 5'- GGC CAT CCA GAG GAG CAT AG -3'
	Reverse: 5'- TTT CTC CAT GCT GCT GTT GAA -3'
IFN-β	Forward: 5'- ACCCGAGCAGCAGTCTCAAG -3'
	Reverse: 5'- ATCCAGTCTCAGAGGCATCAGC -3'
IL-6	Forward: 5'- CCT GAA AGC ACT TGA AGA ATT CC -3'
	Reverse: 5'- GGT ATG CTA AGG CAC AGC ACA CT -3'
IL12p40	Forward: 5'- AAT GCG AGG CAG CAA ATT ACT C -3'
	Reverse: 5'- CTG CTC TTG ACG TTG AAC TTC AAG -3'
IFIT3	Forward: 5'- AGC AGC CTT TTC TTG ACA GC -3'
	Reverse: 5'- ACC ATT CCG CAA CAC ACT TC -3'
CXCL10	Forward: 5'- GCC ATT CAT CCA CAG TTG ACA -3'
	Reverse: 5'- CAT GGT GCT GAC AGT GGA GTC T-3'
IL-1β	Forward: 5'- GGC TGA TGC TCC CAT TCG -3'
	Reverse: 5'- CAC GAG GCA TTT CTG TTG TTC A-3'
IL-1RI	Forward: 5'- AGTTGTCACTCCTGTGCCCC -3'
	Reverse: 5'- TCATATTCTCCATCTGCGTTGTTC -3'
IL-1RII	Forward: 5'- ATGCAAAGGGCCAGGTTCCG -3'
	Reverse: 5'- GAGGGCACCTCAGAGTCACG -3'
CSF1	Forward: 5'- TGACACAGGCAATAAGGAGCA -3'
	Reverse: 5'- AGAGATAGTCTTGTGTGCCCAG -3'
CSF2	Forward: 5'- TCAGTGGGAGTCTGGCGAAC -3'
	Reverse: 5'- TGGTGGCTAATTCCAGCCCC -3'
TNF-α	Forward: 5'- GGA GTG GCT GAG CCA TCG T -3'
	Reverse: 5'- AGC TGG TTG TCT TTG AGA GAC ATG-3'
IRF1	Forward: 5'- GGC ATA CAA CAT GTC TTC ACG -3'
	Reverse: 5'- GCT ATG CTT TGC CAT GTC AA -3'
IRF2	Forward: 5'- AAT GCC TTC AGA GTG TAC CG -3'
	Reverse: 5'- TGT TCA CCG TAC TAT CCA CTT CAT-3'
CCL17	Forward: 5'- CGA GTG CTG CCT GGA GAT C-3'
	Reverse: 5'- TGA TGG CCT TCT TCA CAT GC-3'
CCL22	Forward: 5'- CGC GTA GTG AAG GAG TTC TTC-3'
	Reverse: 5'- TCT TCA CCA GGC CAG CTT A-3'
IL-1RA	Forward: 5'- GCTGCCCGGTGTTCCCTTAT -3'
	Reverse: 5'- TCTGAAGCCATGGGAGAGCG -3'
IL-2RA	Forward: 5'- AAA GCA AGC TAC ACC TAA CCC-3'
	Reverse: 5'- GCC TTG TAT CCT TGA ATG CG-3'
CTSK	Forward: 5'- TGCCTCCCTCCCTTATCCGA -3'
	Reverse: 5'- TTGGCAGGAGGCTCACAGTC -3'
MMP9	Forward: 5'- GGGACGCTCCTATTTGGCCT -3'
	Reverse: 5'- ACCAGCGGTAACCATCCGAG -3'
RANKL	Forward: 5'- CAGCCCTCTCCACGAGGTTT -3'
	Reverse: 5'- CCTCCAGCCACTAAGGACGG -3'
RANK	Forward: 5'- AGAGGGTAGCAGTTGTCCGC -3'
	Reverse: 5'- CTGGGGTAATGGTCCGCACT -3'
OPG	Forward: 5'- TCCGTGAAGCAGGAGTGCAA -3'
	Reverse: 5'- CTGGGGGACAGCTCCTATGC -3'
CSF1R	Forward: 5'- GTCGTCAAGGGCAATGCTCG -3'
	Reverse: 5'- CACCAGGATGCCTGGGTAGG -3'
BGLAP	Forward: 5'- TCTGACAGAGGTACTGCCTTCG -3'
	Reverse: 5'- GCGTTCTGGAGGCCGATTTG -3'

RUNX2	Forward: 5’- GAACCACAAGTGCGGTGCAA -3’
	Reverse: 5’- GGGCACTCACTGACTCGGTT -3’
ALP	Forward: 5’- ATGGACCAGGCCATCGGAAG -3’
	Reverse: 5’- CACCATGGGAGCCAGACCAA -3’
RPL18	Forward: 5’- GTT TATGAGTCGCACTAACCG -3’
	Reverse: 5’- TGTTCTCTCGGCCAGGAA-3’
RdRp (SARS-Cov-2)	Forward: 5’- CGCATACAGTCTTRCAGGCT -3’
	Reverse: 5’- GTGTGATGTTGAWATGACATGGTC-3’
	Probe 5’- FAM-TTAAGATGTGGTGCTTGCATACGTAGAC-lABkFQ -3’

Supplementary table 2. Primers used for RT-qPCR analysis of mRNA expression in murine cells

Genes	Primer sequences
CTSK	Forward: 5'- CTCGGCGTTTAATTTGGGAGA -3'
	Reverse: 5'- TCGAGAGGGAGGTATTCTGAGT -3'
IL-1RI	Forward: 5'- GTGCTACTGGGGCTCATTTGT -3'
	Reverse: 5'- GGAGTAAGAGGACACTTGCGAAT -3'
IL-1RA	Forward: 5'- GCTCATTGCTGGGTACTTACAA -3'
	Reverse: 5'- CCAGACTTGGCACAAGACAGG -3'
MMP9	Forward: 5'- CTGGACAGCCAGACACTAAAG -3'
	Reverse: 5'- CTCGCGGCAAGTCTTCAGAG -3'
TNF-α	Forward: 5'- CAGGCGGTGCCTATGTCTC -3'
	Reverse: 5'- CGATCACCCCGAAGTTCAGTAG -3'
IFN-γ	Forward: 5'- GCCACGGCACAGTCATTGA -3'
	Reverse: 5'- TGCTGATGGCCTGATTGTCTT -3'
IL-6	Forward: 5'- CTGCAAGAGACTTCCATCCAG -3'
	Reverse: 5'- AGTGGTATAGACAGGTCTGTTGG -3'
IL-10	Forward: 5'- CTTACTGACTGGCATGAGGATCA -3'
	Reverse: 5'- GCAGCTCTAGGAGCATGTGG -3'
NOS2	Forward: 5'- GTTCTCAGCCCAACAATACAAGA -3'
	Reverse: 5'- GTGGACGGGTCGATGTCAC -3'
ALP	Forward: 5'- GTCATCATGTTCCTGGGAGA -3'
	Reverse: 5'- GGCCCAGCGCAGGAT -3'
RUNX2	Forward: 5'- GACTGTGGTTACCGTCATGGC -3'
	Reverse: 5'- ACTTGGTTTTTCATAACAGCGGA -3'
BGLAP	Forward: 5'- CAATAAGGTAGTGAACAGAC -3'
	Reverse: 5'- CTTCAAGCCATACTGGTCT -3'
OPG	Forward: 5'- ACCCAGAAACTGGTCATCAGC -3'
	Reverse: 5'- CTGCAATACACACACTCATCACT -3'
CSF1	Forward: 5'- GTGTCAGAACACTGTAGCCAC -3'
	Reverse: 5'- TCAAAGGCAATCTGGCATGAAG -3'
RANKL	Forward: 5'- CAGCATCGCTCTGTTCCTGTA -3'
	Reverse: 5'- CTGCGTTTTCATGGAGTCTCA -3'
PTGES	Forward: 5'- GGATGCGCTGAAACGTGGA -3'
	Reverse: 5'- CAGGAATGAGTACACGAAGCC -3'
COX2	Forward: 5'- TTCAACACACTCTATCACTGGC -3'
	Reverse: 5'- AGAAGCGTTTGCGGTACTCAT -3'
RANTES	Forward: 5'- TTTGCCTACCTCTCCCTCG -3'
	Reverse: 5'- CGACTGCAAGATTGGAGCACT -3'
IL-22	Forward: 5'- ATGAGTTTTTCCCTTATGGGGAC -3'
	Reverse: 5'- GCTGGAAGTTGGACACCTCAA -3'
IL-23	Forward: 5'- CAGCAGCTCTCTCGGAATCTC -3'
	Reverse: 5'- TGGATACGGGGCACATTATTTTT -3'
GAPDH	Forward: 5'- AGGTCGGTGTGAACGGATTTG -3'
	Reverse: 5'- TGTAGACCATGTAGTTGAGGTCA -3'
